# Evaluation of the treatment guidelines, practices and outcomes of complicated severe acute malnutrition in children aged 0-59 months in sub-Saharan Africa: a study protocol for the SAMAC study

**DOI:** 10.11604/pamj.2020.36.241.19584

**Published:** 2020-08-04

**Authors:** Janet Adede Carboo, Martani Lombard, Cornelia Conradie, Robin Claire Dolman, Cristian Ricci

**Affiliations:** 1 Centre of Excellence for Nutrition, North-West University, Potchefstroom, South Africa,; 2 Pediatric Epidemiology, Department of Pediatrics, Medical Faculty, Leipzig University, Leipzig, Germany

**Keywords:** Sub-Saharan Africa, children, severe acute malnutrition, medical complications, treatment guidelines, treatment practices, mortality

## Abstract

**Introduction:**

in hospitals across Africa, the case fatality rates of severe acute malnutrition (SAM) have remained consistently high (over 20%), despite the existence of the WHO treatment guideline. This has been attributed to inconsistencies in the implementation of the WHO treatment guidelines in sub-Saharan African countries. In spite of this awareness, the SAM treatment guidelines adopted by various sub-Saharan African countries and hospitals are unknown. Knowledge of the exact treatment practices employed in the management of SAM in different hospitals in sub-Saharan Africa is not known. This study aims to investigate the admission criteria, in-patient treatment guidelines and practices and outcomes of complicated SAM in sub-Saharan African children aged 0-59 months.

**Methods:**

this is an observational study which involves the extraction of admission, treatment and discharge data from the medical records of infants and children aged 0-59 months diagnosed and treated for complicated SAM in sub-Saharan Africa. This information is being used to develop a comprehensive database on the treatment of complicated SAM across sub-Saharan Africa. Information on the national and hospital guidelines for the treatment of complicated SAM is also collected.

**Results:**

results of this study will serve as a useful resource on the true reflection of the treatment of complicated SAM across sub-Saharan Africa and will provide valuable information for optimising SAM treatment.

**Conclusion:**

in order to advocate best practice and reduce SAM-related mortality in sub-Saharan Africa, the identification of the different diagnostic and treatment methods and respective outcomes across different hospitals and countries is imperative.

## Introduction

Undernutrition remains a public health burden and has gained global significance. It is captured in a number of the United Nations’ sustainable development goals, especially goal 2, which aims at ending hunger, achieving food security, improving nutrition and promoting sustainable agriculture [1]. Child undernutrition causes up to 45% of all deaths in children younger than the age of five years. This accounts for more than three million child deaths annually [2]. Other than the morbidity and mortality risk, undernutrition also contributes to impaired intellectual development, suboptimal work capacity as an adult, as well as an increased risk for disease in adulthood [2]. A reduced work capacity generally contributes to a reduced income potential leading to an intergenerational cycle of poverty [3]. Severe acute malnutrition (SAM) is an extreme form of nutrient deprivation as a result of inadequate dietary consumption, malabsorption of ingested nutrients and/or acute or chronic disease or infections, which increases nutrient requirements while promoting nutrient loss and catabolism over a short period of time [4]. It is characterised by a weight-for-length/height z-score (WHZ) of <-3 standard deviation, mid-upper arm circumference (MUAC) <115mm, and/or the presence of bilateral pitting oedema [5]. It is also accompanied by profound organ dysfunction, physiologic and metabolic imbalances.

According to the 2019 United Nations Children’s Fund (UNICEF), World Health Organization (WHO) and the World Bank estimates, approximately 49 million and 17 million children in low and middle-income countries were affected by wasting and severe wasting, respectively, in 2018 [6]. Out of this number, 13.3 million of the wasted and 4 million of the severely wasted children are found in sub-Saharan Africa [6]. Aside from these estimates, the global prevalence of malnutrition-related oedema in children remains uncertain due to the lack of its assessment in large nutritional surveys [7]. Hence, the actual burden of acute malnutrition in children worldwide may be underestimated [7]. With the introduction of the community-based management of SAM, children with SAM who have a good appetite and are devoid of any medical complications are treated as out-patients. However, those who have additional medical complications such as severe infections, severe dehydration and shock are classified as complicated SAM and require initial hospitalisation and stabilisation prior to out-patient care [5]. The WHO has hence developed clinical guidelines [8,9] for the treatment of children with SAM. The guidelines for in-patient management are set out as simple and specific instructions that aim to restore metabolism through the correction of electrolyte imbalance, reversal of metabolic abnormalities, restoration of organ function and the provision of nutrients to ensure catch-up growth [8].

In consideration of the intense health burden caused by SAM, the WHO, recommends the use of MUAC as a screening tool for early detection of SAM at community level and the independent use of either MUAC or WHZ and/or the presence of oedema for the diagnosis of SAM at the hospital [5]. However, in recent times, there has been a move to promote the lone use of MUAC for the diagnosis of SAM, owing to some studies that have reported the superiority of MUAC over WHZ in the identification of malnourished children at highest risk of mortality [10,11]. This assertion has however been rebutted in a recent study by Grellety and Golden [12]. In the face of the on-going controversy over the preferred SAM diagnostic tool, knowledge of the actual practices regarding the identification and diagnosis of SAM among children in hospitals across Africa is limited. MUAC and WHZ have however, been reported to correlate poorly and identify different groups of malnourished children [13] and may, therefore, influence response to treatment. In sub-Saharan Africa, where malnutrition-related mortality is at its highest [14], the actual practices regarding the identification and diagnosis of SAM are critical. Timely identification and appropriate diagnosis are essential for early initiation of treatment, which minimises the risk of complications and worse outcomes [15].

Furthermore, in recent years, it has been acknowledged that there is a growing prevalence of SAM in infants younger than 6 months [16]. However, evidence regarding the classification and management of SAM in infants younger than six months is still limited [17]. Acute malnutrition in this age group was traditionally overlooked, but in 2010, the management of acute malnutrition in infants (MAMI) report was the first to emphasize this matter [18]. Much international interest led to the dedication of a separate chapter on this in the WHO 2013 document “updates on the management of severe acute malnutrition in infants and children” [5]. This chapter emphasizes the rather limited empirical evidence to inform necessary recommendations and highlighted this as a priority area for future research [5]. According to WHO, with the appropriate implementation of its in-patient treatment guidelines for SAM, mortality should be reduced to <5% [19]. However, in a recent systematic review, it was indicated that despite the implementation of the guidelines, children admitted for complicated SAM due to severe infection typically have a case fatality of 12% to more than 20% [20].

The 2013 WHO update on the management of SAM in children under five years, provides an appraisal of the evidence used to produce the recommendations and reports a paucity of research in the field. It, therefore, urges for more research on the treatment of SAM and its related conditions, especially in infants less than 6 months of age [5]. This is mainly due to a limited number of studies evaluating the diagnostic criteria and investigating existing and other methods of the nutritional and medical management of SAM. Regardless of the WHO treatment guidelines, discrepancies in treatment practices and protocols between different sub-Saharan African countries, regions, hospitals and even health care personnel are recorded [21]. Little is, however known about how these different clinical practices in the management of SAM affect treatment outcomes, as well as how this compares with the guidelines proposed by the WHO. Consequently, the *Severe Acute Malnutrition in African Children* (SAMAC initiative-collaboration-group) was initiated to investigate and compare the admission criteria, in-patient treatment guidelines and practices and treatment outcomes of complicated SAM in sub-Saharan African children aged 0-59 months.

**Figure 1:**
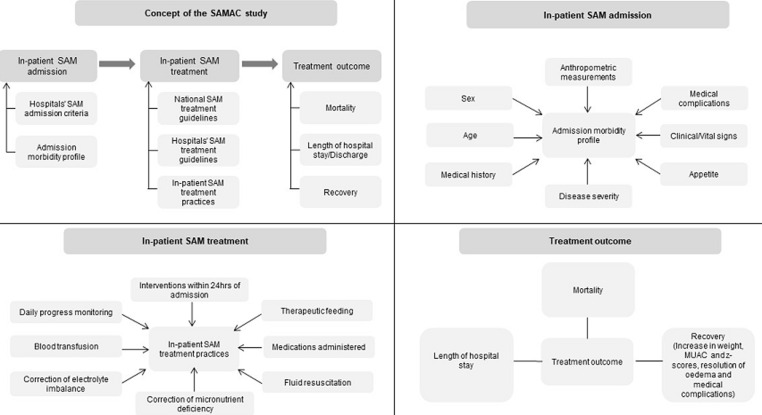
describes the concept of the SAMAC study and provides details of the variables and outcomes of interest of the study

**Aims of the study:** the aims of this study are: to identify different in-patient SAM admission criteria across selected sub-Sahara African countries and hospitals; to compare different treatment/clinical practices with local and WHO guidelines for the in-patient treatment of complicated SAM; to investigate the determinants of treatment outcomes such as mortality, recovery and length of hospital stay. A secondary aim of the study is to develop and validate a SAM severity score based on the association between admission profile and outcomes and to develop a comprehensive standardised database for capturing data on treatment procedures of complicated SAM in children 0-59 months across sub-Saharan Africa. Other factors that are being investigated to achieve the aims of the study are shown in Figure 1.

## Methods

**Study design:** this is an observational multi-country and multi-hospital study. It is based on the review and collection of diagnosis and treatment data from the medical records of children and infants aged between 0 and 59 months, diagnosed, hospitalised and treated for SAM after January 2013 when the latest WHO treatment update document was released. Briefly, data being collected include all medical and clinical information presented at admission, during the time of hospital stay and at discharge from in-patient care as documented by the clinicians in the medical records. Information on the different national or provincial SAM diagnosis and treatment guidelines is collected in consultation with the national and provincial departments of health where applicable. Information on the treatment guidelines used in the various malnutrition units of the selected hospitals is also collected in consultation with the heads of the units.

**Figure 2:**
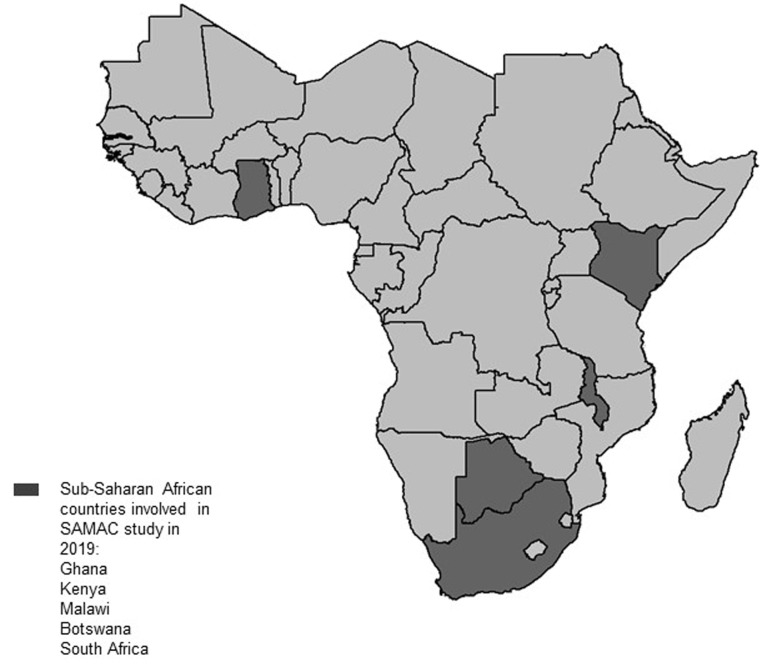
highlights the countries currently included in the SAMAC study: Ghana, Kenya, Malawi, Botswana and South Africa

**Setting:** currently, five sub-Saharan African countries (South Africa, Ghana, Botswana, Malawi and Kenya) are included in the study (Figure 2). However, negotiations with other interested sub-Saharan Africa countries are underway. Selected hospitals admitting and treating infants and children for complicated SAM from each country are included in the study. The implementation of the study is coordinated by the Centre of Excellence for Nutrition of the North-West University through collaborations with local coordinators in the various countries and hospitals. This study has been approved by the Health Research Ethics committee of the North-West University, South Africa (NWU-00063-17-A1). Data from medical records of infants and children aged between 0-59 months who are admitted and treated for complicated SAM (regardless of the diagnostic criteria used) at any of the included hospitals after January 2013 are reviewed and extracted. The medical records are screened using a pre-designed screening tool based on the inclusion and exclusion criteria. The inclusion criteria are: infants and children aged 0-59 months diagnosed, hospitalised and treated for SAM after January 2013 in the selected hospitals; and infants and children admitted primarily for other medical conditions but who are diagnosed and treated for SAM during the hospital stay. The exclusion criteria include: children (0-59 months) diagnosed with SAM and additional metabolic, neurodevelopmental or any growth disorders that are predispositions to feeding difficulties or requirement of specialised feeding; infants younger than 6 months of age diagnosed of SAM but who were born premature or with a low birth weight (<2.5kg) and 3) children (0-59 months) diagnosed with SAM but with incomplete medical information, whose treatment outcomes cannot be ascertained.

**Data collection:** the medical records are preliminarily screened for inclusion. Records that meet the inclusion criteria are entered into a registry to avoid duplication. Data is extracted using a valid structured data extraction tool (supplementary material) adapted and developed from three published WHO documents on improving the in-patient management of SAM to ensure content validity [22]. Data is extracted by trained research assistants and daily cross-checked during the capturing to ensure quality. Table 1 summarises the data of interest extracted and at which time point during the hospital stay. Unavailable or missing data is acknowledged and recorded as such. For children who are admitted multiple times for complicated SAM, data of each readmission is extracted and noted as such.

**Table 1:** summary of data extracted in SAMAC study

	First 24 hours of admission	During hospital stay (>24 hours of admission)	Discharge from in-patient care
Demographic data	X		
Medical history	X		
Anthropometry	X	X	X
Clinical and vital signs	X	X	X
Medical complications	X	X	X
Bed chart information		X	X
Appetite	X	X	X
Nutritional treatment	X	X	X
Micronutrients/electrolyte/trace element administration	X	X	X
Fluid administration and blood transfusion	X	X	
Biochemical information		X	
Hydration status	X	X	X
Medications	X	X	X
Health professionals involved with treatment	X	X	X
Daily progress monitoring		X	X
Referral for out-patient care			X
Nutritional counseling of caregiver			X

### General characteristics at admission

**Demographic information:** this includes the country of residence, hospital and provincial location, age, sex, whether or not the patient was referred to the treatment hospital and name of referring hospital. The date of birth is recorded and converted to age in months.

**Health status and anthropometry at admission (status of the child within the first 24 hours of admission):** all information on the health and physical status of the malnourished child within 24 hours of admission is recorded. This entails; time and date of admission, anthropometric measurements (weight, height, mid-upper arm circumference, weight-of-height z-scores), clinical signs (irritability, shock, cold peripheries, dermatosis, eye signs and signs of dehydration, oedema grade), vital signs (temperature, heart rate, capillary refill time, blood glucose levels e.t.c), medical complications presented, HIV and TB status, administration of intravenous fluid (type, volume and duration), rehydration solutions, correction of hypoglycaemia within 30 minutes of admission and whether or not feeding was commenced within 30 minutes of admission. This data will provide information on the morbidity characteristics of the severely malnourished child at admission and the immediate treatment interventions administered to correct emergency life-threatening complications such as unconsciousness, convulsion, hypoglycaemia, hypothermia, severe dehydration and shock presented [23]. The correct identification of presenting complications and prompt interventions and stabilisation within the first few hours of admission are critical to the survival of a child with complicated SAM [24,25].

### Information on daily health monitoring during hospital stay (status after the first 24 hours of hospital stay)

**Bed chart information (vital signs during hospital stay):** this refers to vital signs recorded after 24 hours of admission. Data on the daily body temperature (monitored 4 hourly), pulse and blood glucose level per day are recorded. The purpose of this information is to monitor the resolution or development of hypothermia, fever, hypoglycaemia, as well as signs of shock during the course of the treatment [26].

**Dehydration:** information on the frequency and consistency of stools, frequency of vomiting per day and urine output are recorded daily throughout the period of hospital stay. Other clinical signs of interest are skin recoil and sunken eyes. This is to monitor and review the resolution or development of diarrhoea and vomiting in response to the feeds and medication as well as monitor signs of overhydration. All fluids administered, including both oral and intravenous, are recorded together with the type, rate and duration of administration. This information is very important in the management of SAM as WHO guidelines on fluid management in SAM are extremely cautious, owing to the postulation of probable fluid overload and cardiac failure as a result of a compromised cardiovascular system [5]. Further, the optimal fluid management of malnourished children with severe dehydration and shock is unknown [27].

**Biochemical information:** all data on biochemical measurements during hospital stay including full blood count, blood electrolytes, urea, creatinine and albumin are extracted. This provides information on how deranged physiological and metabolic parameters that usually occur with SAM are corrected or worsened in response to treatment. All severely malnourished children have an electrolyte imbalance, especially sodium, potassium and magnesium [23]. Excess body sodium may exist even though plasma sodium may be low [23]. Hence, information on how these imbalances are resolved during treatment is important for recovery. Data on renal and liver function are also recorded as these may be compromised with SAM [5].

**Nutritional treatment:** information on the date and time when feeds were commenced and given is recorded from the feeding chart in the medical records. The type of starter and transitional feeds, total volume prescribed per day, route of administration, rate and the actual volumes consumed are also recorded. Similarly, information on other foods prescribed and consumed while the patient was on either starter or transition feeds are also recorded.

**Micronutrient, electrolyte and trace elements:** all severely malnourished children have micronutrient and vitamin deficiencies [5]. Therefore, micronutrient supplementation forms an important part of treatment. Some micronutrients of importance emphasised by the treatment guideline include vitamin A, folic acid, zinc, copper and iron [26]. Hence, information on the prescription, administration date and dosage of micronutrients and vitamins particularly vitamin A, folic acid, iron, multivitamin, potassium chloride and zinc administered during the treatment period are extracted. This is reviewed in conjunction with whether or not the patient was being fed ready-to-use therapeutic food, as it already contains some vitamin A, folic acid, zinc and copper.

**Medical treatment:** medications prescribed and administered from the first to the last day of hospital admission are recorded. This information includes the names of all medications, especially antibiotics, the reason for prescription, date, dosage and route of administration. Information on antiretroviral therapy and blood transfusion where applicable during hospital stay is also extracted.

**Daily progress evaluation:** as part of the daily health monitoring, daily progress made in response to treatment is collected. Data on daily anthropometric measurements, oedema grade, appetite, clinical signs and complications are recorded. This information is to monitor the rate of recovery and response to treatment.

**Discharge information (discharge from in-patient to out-patient care):** discharge information including date of discharge from in-patient care, clinical signs, clinical wellness, persisting medical complications, appetite, oedema grade, anthropometric measurements, referral to an out-patient facility for continuous care and rehabilitation, whether or not caregiver was given health and nutrition education to prevent relapse and the different health professionals who cared for the patient throughout hospital stay are also recorded.

**Mortality:** the date of death and probable cause of death as documented by the clinician in the medical records is extracted for cases who died.

**Treatment outcome:** the treatment outcomes of interest in this study include recovery (resolution of oedema and medical complications), weight gain, MUAC and changes in z-scores, length of hospital stay and mortality.

**Sample size calculation:** two sets of power calculations were conducted to define the adequate sample size by hospital. A first power calculation was conducted considering mortality as the main outcome. To this aim, a multivariate adjusted Cox regression with a type-I error of 5% (α=0.05) was considered. In this first power calculation, we considered a hazard ratio (effect size) of 1.5 with an expected 10% number of events and a residual R-square among adjusting covariates of 0.15. According to this power calculation, a required statistical power of 90% (β<0.1) would require at least 750 subjects. A second power calculation in the framework of generalized linear model was performed for continuous outcomes of interest. This second power calculation was performed considering a multivariate adjusted model having at least ten covariates. For this second power calculation, we considered a medium Cohen f^2^ standardized effect size (f^2^=0.25). A power calculation considering post-hoc analyses for group by group comparison was performed by means of a t-test aimed to detect a medium Cohen’s d effect size (d=0.5) with type-I error adjusted with Bonferroni correction [28]. The above reported sample size of 750 subjects by centre defined for the Cox model was sufficient also for this second scenario applicable to continuous outcomes. According to a likely missing rate of about 20%, we defined that the ideal sample size by centre would not be less than 1000 individuals. Power calculations for the Cox model were performed by the proc power of the SAS statistical software vers 9.4. The power calculation considering Cohen effect sizes were performed using the G*power software version 3.1.4.

### Data management and analysis

**Data management:** data is extracted under strict confidential measures, anonymised and is accessible to only research assistants and supervisors during the data extraction process. The extracted data is captured and saved into a password-protected Microsoft Access 2016 database with the password known to only persons responsible for data capturing and the principal investigators. Data capturing is managed and co-ordinated by a dedicated data manager who checks all captured data for correctness. The finalised database will be stored in a secured electronic system and backed-up on a dual-core electronic processor.

**Data analysis:** centre characteristics will be described by country using position and dispersion indexes according to variable skewness. Statistical analysis will be conducted hierarchically considering hospitals as analysis unit, then results from hospitals will be pooled by country. To this aim, a multivariate-adjusted analyses adjusted for main confounders will be used to estimate the risk of mortality for different treating protocols and length of stay by centre. Time to event analyses will be conducted using different approaches. A multivariate Cox model adjusted for main confounders will be used when the assumption of hazard proportionality is respected. Otherwise, the accelerated failure model will be considered. Supplementary evaluations will be performed considering stratification according to age (<6 months, 6-11 months, 12-23 months and 24-59 months). This stratification is to observe the variations in the response to treatment within the different age groups owing to their varied nutrient requirements and growth rate. Relative risk of mortality obtained by centre will be meta-analysed using a random effect model and heterogeneity between centres will be evaluated by country using the Cochrane Q test. Finally, results by country will be compared using a linear fixed effect meta-regression approach. Secondary outcomes will be investigated using multivariate generalized linear models adjusted for potential confounders. Statistical analysis will be performed using the SAS software package vers.9.4 (PROC PHREG, PROCLIFEREG, PROC GLM, PROC MIXED). Pooled risk estimates of mortality by country, heterogeneity estimation and meta-regression to compare country performances will be performed by STATA vers.15 (METAN and METAREG functions). When a sufficient coverage of sub-Saharan Africa is reached, geospatial analyses and representations by maps will be undertaken to improve results dissemination. This further step will be conducted with STATA vers 15 (SPMAP and related functions).

**In-patient SAM treatment database:** the valid data extraction file developed for this study is accompanied by a large database that is linked to the extraction file. The database was developed using Microsoft Access version 2016. It consists of multiple data sheets, each for capturing data on the different aspects of in-patient treatment of complicated SAM from admission to discharge or death. It consists of a well-structured, user-friendly data imputation interface which was adapted from three published WHO documents on improving in-hospital treatment. It contains different datasets on patients’ demographics, admission characteristics, therapeutic feeding, medications, micronutrient and electrolyte correction, blood transfusion, rehydration, daily progress, health professionals involved in care and discharge information. It also reports on mortality, length of stay and re-admission.

## Results

Results of this study will serve as a useful database, which will be a true reflection of the treatment of complicated SAM across various hospitals in sub-Saharan Africa. This resource will provide valuable information for influencing policy and optimising complicated SAM treatment across Africa.

## Discussion

The importance of optimal strategies in the management of complicated SAM and their role in the survival of the severely malnourished child has been documented and cannot be overemphasized [5]. Complicated SAM is considered a medical emergency and is associated with an increased risk of death [2]. In hospitals across Africa, the case fatality rates of SAM have remained consistently high (over 20%) [20], despite the existence of the WHO treatment guideline. This has been attributed to inconsistencies in the implementation of the WHO treatment guidelines in sub-Saharan African countries [21]. In spite of this awareness, the SAM treatment guidelines adopted by various sub-Saharan Africa countries and hospitals is not known. Even more importantly, knowledge of the exact treatment practices employed in the management of SAM in different hospitals in sub-Saharan Africa is not known. Again, the effect of the different practices and guidelines on treatment outcomes has not been investigated and compared. In order to advocate best practice and reduce SAM mortality, the identification of the different diagnostic and treatment methods and their respective outcomes across different hospitals and countries is imperative. This study is novel due to its large coverage and data being obtained from different countries across sub-Saharan Africa on the treatment guidelines and practices of complicated SAM and the probable variations in methods and outcomes. The large multi-country database will serve as a useful resource on the true reflection of the in-patient treatment of complicated SAM across sub-Saharan Africa and will provide valuable information and in-depth knowledge of the accompanying co-morbidities via the use of statistical modeling.

It is therefore anticipated that this study will successfully contribute to the available data and resource used for policy development on the optimal management of children with complicated SAM. This study also is a simple and cost-effective approach to obtain large valuable data on the treatment of complicated SAM across sub-Saharan Africa. A limitation of this study is that there is no control over the quality of anthropometric measurements and exposures at admission and discharge. However, measurements that look inaccurate are crosschecked for confirmation of accuracy. This notwithstanding, our study has great benefits. Although there are several internationally recognised databases on SAM, none provides information on the in-patient admission criteria, actual treatment practices, daily response to treatment and outcomes of complicated SAM in hospitals across sub-Saharan Africa as our study will do. This study will, therefore, provide valuable information on the differences in the criteria for in-patient admission, treatment guidelines and practices and outcomes of complicated SAM treatment in infants and children in sub-Saharan Africa. This will also identify treatment practices and facilities that require changes, as well as those that need to be upheld and emulated. This will thus, have a direct impact on the treatment protocols of all infants and children diagnosed with SAM, especially in terms of admission criteria, medical nutrition therapy and fluid management.

## Conclusion

To advocate best practice and reduce SAM-related mortality in sub-Saharan Africa, the identification of the different diagnostic and treatment methods and their respective outcomes across different hospitals and countries is imperative for policy development and advancement in SAM treatment.

### What is known about this topic

- Complicated SAM is associated with high mortality;
- The high SAM mortality has been attributed to inconsistencies in the implementation of WHO treatment guidelines across hospitals and faulty management of complications.

### What this study adds

- This study will provide valuable information on the actual SAM treatment guidelines and practices employed in the treatment of SAM in selected hospitals across sub-Saharan Africa and the associated outcomes;
- It will also generate a large database on the details of SAM treatment (including medical and nutritional treatment strategies, fluid management, micronutrient deficiencies and electrolyte imbalance correction) across sub-Saharan Africa. This will be a useful resource for stakeholders in SAM management and policy development.
